# Removal of proprioception by BCI raises a stronger body ownership illusion in control of a humanlike robot

**DOI:** 10.1038/srep33514

**Published:** 2016-09-22

**Authors:** Maryam Alimardani, Shuichi Nishio, Hiroshi Ishiguro

**Affiliations:** 1 Advanced Telecommunications Research Institute International, Kyoto, 619-0288, Japan; 2 Department of General Systems Studies, Graduate School of Arts and Sciences, The University of Tokyo, Tokyo, 153-8902, Japan; 3 Department of System Innovation, Graduate School of Engineering Science, Osaka University, Osaka, 560-8531, Japan

## Abstract

Body ownership illusions provide evidence that our sense of self is not coherent and can be extended to non-body objects. Studying about these illusions gives us practical tools to understand the brain mechanisms that underlie body recognition and the experience of self. We previously introduced an illusion of body ownership transfer (BOT) for operators of a very humanlike robot. This sensation of owning the robot’s body was confirmed when operators controlled the robot either by performing the desired motion with their body (motion-control) or by employing a brain-computer interface (BCI) that translated motor imagery commands to robot movement (BCI-control). The interesting observation during BCI-control was that the illusion could be induced even with a noticeable delay in the BCI system. Temporal discrepancy has always shown critical weakening effects on body ownership illusions. However the delay-robustness of BOT during BCI-control raised a question about the interaction between the proprioceptive inputs and delayed visual feedback in agency-driven illusions. In this work, we compared the intensity of BOT illusion for operators in two conditions; motion-control and BCI-control. Our results revealed a significantly stronger BOT illusion for the case of BCI-control. This finding highlights BCI’s potential in inducing stronger agency-driven illusions by building a direct communication between the brain and controlled body, and therefore removing awareness from the subject’s own body.

For centuries, philosophers quested to find the relationship between the *body* and the experience of *self*. Yet, our understanding of the mechanism through which the human mind recognizes a certain body as the self remained limited until recently, when Botvinick and Cohen introduced the rubber hand illusion (RHI)^1^. They showed that touches on a rubber hand placed in front of a subject along with the simultaneous touches on the subject’s hidden hand could produce a feeling of ownership and displacement toward the seen fake hand as if the rubber hand became a part of the subject’s own body. This was the first empirical evidence that confirmed the sense of self is not coherent and under specific conditions one can experience other bodies as a part or whole self. The discovery of RHI initiated a new research area, where researchers probed conditions and brain mechanisms responsible for the induction of similar illusions^1^,^2^,^3^,^4^,^5^,^6^,^7^,^8^,^9^. For instance, out-of-body experiences^10^ or body swapping experiments^11^ demonstrate that the illusion can be extended to one’s entire body (whole-body ownership). Other studies reported that not only watching a synchronous touch but also watching a non-body hand^7^,^8^ or a virtual hand^9^ move in synchronization with one’s hidden hand could induce a sense of agency which eventually led to the sensation of ownership for the fake moving hand. Other works revealed that during the illusion induction, a certain level of temporal^12^,^13^,^14^ and anatomical congruency^15^,^16^ between the sensory inputs was crucial for the brain to make the adjustment between the new information and the pre-existing model to recalibrate the body representation and incorporate the new part in the self-body. An extreme example for this is the case of tool studies, where bodily frame and sensations are extended to the tools but the feeling of ownership remains intact^17^. Since body ownership illusions are considered as an outcome of the interaction between the sensory inputs and the internal models of body^7^,^18^,^19^,^20^, identifying the basic principles that govern the induction of these illusions can provide an invaluable tool for understanding the brain processes that modulate the sense of body ownership and experience of self^4^,^21^.

In a similar line of research, we previously reported an illusion of body ownership transfer (BOT) for the operators of a very humanlike android robot^22^,^23^,^24^. We first demonstrated that the operators experienced an illusion of owning the robot’s hand, when they moved their own hand and, inside a head-mounted display (HMD) watched images of the robot’s hand copy their actions in a perfectly synchronized manner^23^. In this experiment, when we delayed the robot’s motions for 1 second (visual feedback delay in HMD), the illusion experienced by the operators became significantly weaker but did not shatter. However, when in a new set of experiments, we implemented a brain-computer interface (BCI) for the operation^24^ and the operators could control the robot just by imagining the movement -without moving their own body, we found out that they could experience a significant BOT illusion even with a rather long delay (about 1 second) in the BCI system. This difference between the outcomes of the two experimental setups (motion-control and BCI-control) left an unanswered question about the effect of the temporal delay on the agency-driven illusions.

From past studies, we already knew that in sensory-driven illusions such as RHI, temporal delays longer than 300 ms^13^ or spatial mismatches^15^ in the visuotactile stimulation put a critical constraint on the intensity of illusion. Also, several studies on the motion-driven illusions reported a dissociation of the two senses of agency and ownership due to temporal asynchrony^25^,^26^ or spatial incongruence^8^,^27^, which led to the interruption of the body ownership illusion even though the subjects felt control over the body. In contrast, in a recent investigation of virtual embodiment, Kokkinara *et al*. introduced a visuomotor adaptation to spatiotemporal mismatches^28^. When the virtual body movement accompanied a velocity or spatial scaling manipulation, the subjects’ perceived body ownership for the virtual agent did not change, although the perceived agency and proprioceptive judgment of the space was affected. These works suggest that agency and ownership can be both plastic to temporal mismatches^26^,^29^ under certain conditions. However, the corresponding mechanism through which the body ownership and agency interacted with the visuomotor temporal delays remained unclear.

In our experiments with the teleoperated robots, the only difference between the two experimental conditions was the presence or absence of subjects’ bodily movements. During the motion-control condition, subjects experienced the BOT illusion by receiving visual feedback from the robot’s body and movement-related proprioceptive feedback from their own actions (Fig. 1a). In the BCI-control condition, however, the application of the BCI system removed the afferent proprioceptive feedback, leaving subjects with the motor intentions and visual feedback of their imagined movements. Therefore, one can say that the difference between the BOT illusions in the two experimental conditions emerged from the contribution of the afferent proprioceptive feedback and the mechanism it interacted with the delayed visual feedback.

Proprioception, the internal sensation of body positioning and movement kinematics, is considered as a primary element in establishing our sense of agency and bodily-awareness during movement^20^. Receptors located in skin, joints and muscles continuously send afferent information that is processed along with the efferent motor commands to make ongoing judgments of position and movement^30^,^31^. In previous studies, agency-driven illusions were induced by synchronizing the motor-related proprioceptive inputs with manipulated visual information^7^,^8^,^9^,^32^. However, in our work with the BCI-operated robots, we showed for the first time that even without receiving such afferent proprioceptive updates, one could experience agency and ownership for a humanlike moving body^24^. This meant that afferent proprioception was not essential to our sense of agency and even in its absence our body-recognition model could be regulated. Nonetheless, it was yet unknown how the proprioceptive inputs interacted with the visual feedback to contribute to the inducement process and how the illusions induced in each experimental condition (motion-control vs. BCI-control) differed from another. Particularly under temporal delay, which was inevitable in both operational interfaces, the role of proprioception and motor commands remained a question. Did the afferent proprioceptive signals regarding movement have an enhancing effect due to the multisensory integration with vision or did they have an inhibiting effect because of the temporal mismatches between sensory inputs at the cognitive levels (Fig. 1a)?

To answer the question above, in this study we particularly focused on the role of proprioceptive afferents in the inducement of agency-driven illusions especially when there is an apparent and perceptible delay in the visual feedback. We compared the intensity of the BOT illusion for the robot operators in two operational sessions: (1) Motion-control: when they performed the motion and a motion-capture (MoCap) system copied their movements to the robot and (2) BCI-control: when they held an image of the motion and a BCI system translated their motor imagery to the robot’s motions. In both sessions, the robot motions were accompanied by a certain amount of delay. We raised the hypothesis that under delayed visual feedback, the direct BCI-control of robot’s body and thus elimination of such mismatching signals as proprioception can raise a stronger BOT illusion.

## Results

Thirty-three subjects participated in our experiment. Each participant performed the following two sessions in a random order.

1. MoCap session: Subjects grasped their own right or left hand to control the robot’s corresponding hand.
2. BCI session: Subjects performed a right or left motor imagery task and controlled robot’s hands without actual motions.

**Figure 1:**
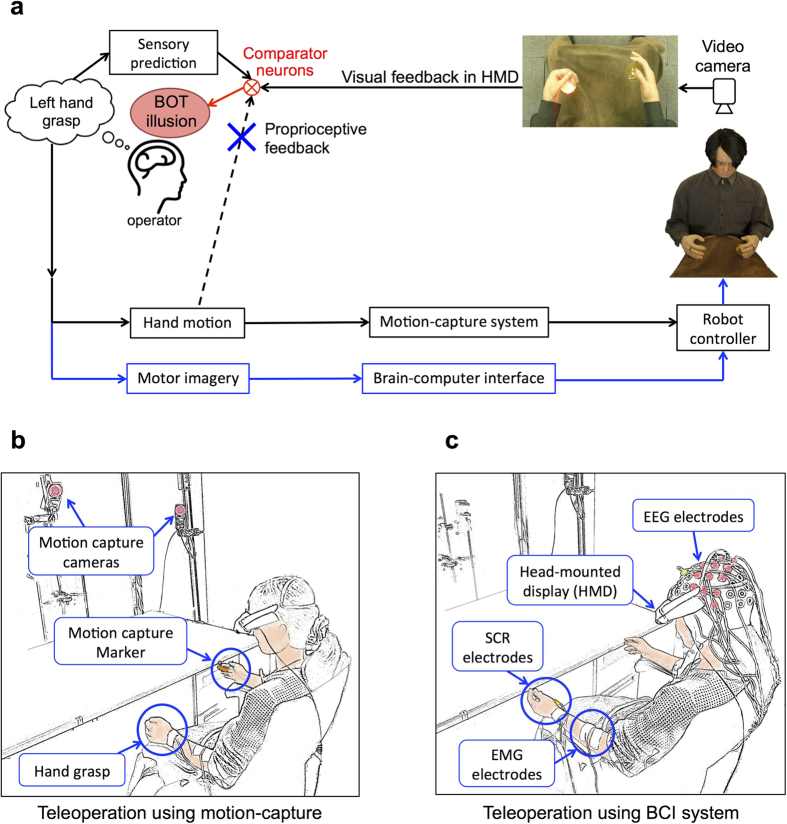
Body ownership transfer (BOT) during teleoperation of a humanlike robot. (**a**) The cognitive model of body recognition suggests that when operators control the robot’s body, the match between the sensory prediction of motor intentions and the sensory feedbacks (the visual feedback from the robot’s body in the head-mounted display and the proprioceptive feedback from the operator’s body) yields an illusion that the robot’s body belongs to the operator (BOT). To investigate the effect of proprioceptive feedback, subjects controlled the robot’s hands using two interfaces; (**b**) a motion-capture system that copied the operator’s grasp to the robot’s hands (with proprioception) and (**c**) a BCI system that translated the subjects’ EEG patterns of motor imagery to robot’s hand motions (without proprioception). In both sessions, skin conductance response (SCR) and EMG signals were recorded and monitored.

**Figure 2:**
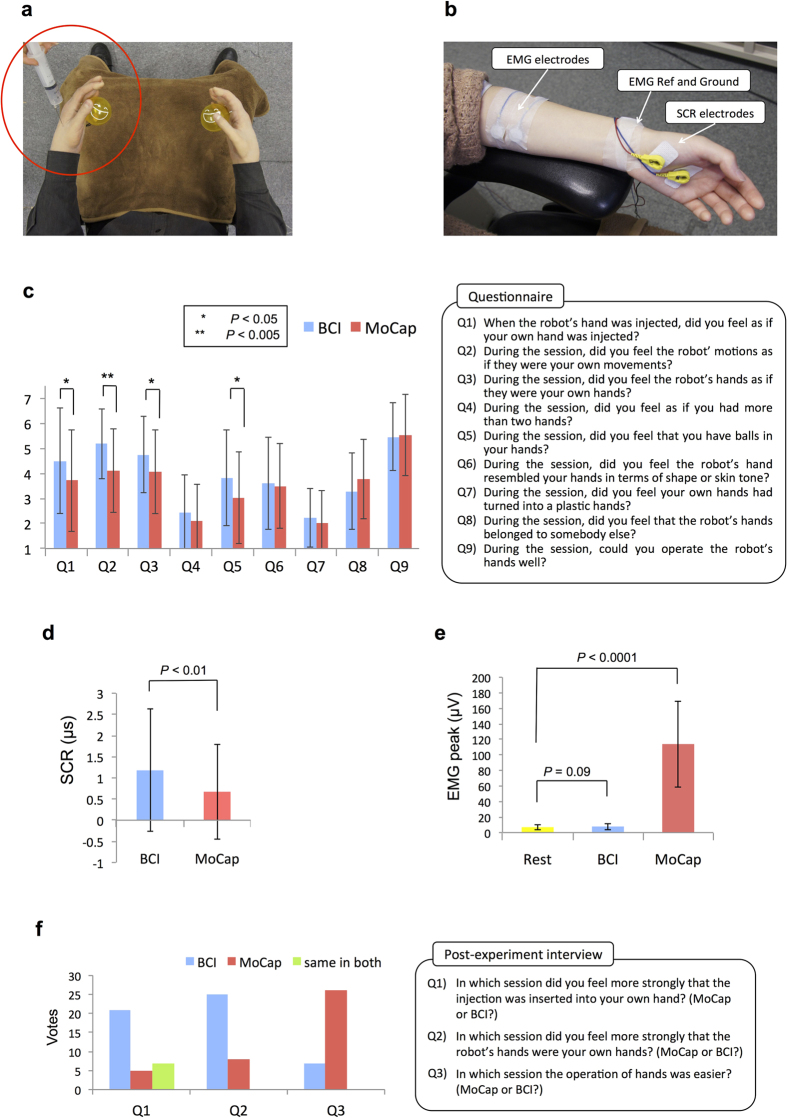
Evaluation results for questionnaire, SCR, EMG and post-experimental interview (**a**) The intensity of BOT illusion was mainly evaluated by measuring subjects’ reaction to a painful stimulus (injection) applied to the robot’s hand at the end of each session. (**b**) EMG electrodes were attached to both left and right arms with the ground and reference electrodes attached to the wrist bones. SCR electrodes were attached to the left palm. (**c**) Subjects scored Q1~Q9 in each BCI and MoCap session based on a 7-point Likert scale, 1 denoting “didn’t feel at all” and 7 denoting “felt very strongly”. Mean values, standard deviations and *p*-values (Wilcoxon Signed-Rank test) of the obtained scores are shown on the graph. Statistical significance was found in Q1, Q2, Q3 and Q5 showing an overall higher BOT illusion in the BCI session. (**d**) SCR peak value, measured immediately after the injection, was assigned as the reaction value. Mean reaction values and standard deviations are plotted. BCI responses revealed a significantly higher reaction to the injection. (**e**) The mean EMG activity measured from the subjects’ arms in each session was averaged per condition and compared to the EMG values in the Rest phase. Results confirmed the absence of movement and unconscious muscle contractions during the BCI session. (**f**) In the post-experimental interview, subjects were asked three questions in which they voted for the session with stronger sensation of injection (Q1), level of ownership (Q2) and task feasibility (Q3). Although MoCap was significantly selected as the easier session, BCI was chosen as the session with higher sensation of injection and ownership.

In the MoCap session, subjects wore markers on their right and left middle fingers. A 3D motion-capture system tracked the marker position and copied the grasp motion to the robot’s hand (Fig. 1b). In the BCI session, brain activities were recorded by 27 EEG electrodes across the sensori-motor area and were classified into two classes of right or left hand motions for the robot (Fig. 1c). In both sessions subjects watched a first-person perspective image of the robot’s motions through a head mounted display (HMD). Two balls, placed inside the robot’s hands, randomly lighted and indicated the hand (either right or left) and the timing for the imagery grasp (Fig. 1a). Subjects practiced the task in a training session before the actual run started. Training was especially longer for the BCI session because BCI performance is often not favorable for novice users. Since the subject’s performance significantly influences the intensity of illusion they experience^33^, their BCI performance was positively biased in both training sessions and control sessions for all subjects in order to remove the negative effect of classifier mis-performance in failed trials (see Methods). Each test session consisted of twenty trials (2 mins and 40 secs) and was terminated by inserting a syringe into the robot’s left hand (Fig. 2a). The evaluation of BOT intensity was made by two measurement methods: 1) A post-session questionnaire that was designed in accordance with RHI questions^1^ and adjusted to the context of motion (Fig. 2c). Subjects orally answered the questions based on a 7-point Likert scale, 1 denoting “didn’t feel at all” and 7 denoting “felt very strongly”. 2) Skin conductance responses (SCR)^3^ (Fig. 2b) that measured subjects’ physiological reaction to the painful stimulus (injection). The SCR peak amplitude within a 6-second interval, 1 second after the appearance of syringe in the participant’s view to 5 seconds after the injection (see Methods), was selected as the reaction value^24^.

The acquired questionnaire scores in each session were averaged and shown in Fig. 2c. A non-parametric Wilcoxon Signed-Rank test was used to compare the questionnaire items as Shapiro-Wilk test rejected normality of Likert scores. Responses to Q1 showed a significantly higher sensation of injection into one’s own hand in the BCI session (M = 4.5, SD = 2.01) than the MoCap session (M = 3.71, SD = 2.03); [BCI > MoCap, *Z* = −2.182, *p* = 0.014]. Results for the agency question Q2 were also significantly higher in the BCI condition (M = 5.18, SD = 1.39) than the MoCap condition (M = 4.11, SD = 1.66); [BCI > MoCap, *Z* = −2.845, *p* = 0.002]. Also, Q3 showed a significantly higher feeling of body ownership for the robot’s hands in the BCI session (M = 4.79, SD = 1.53) than the MoCap session (M = 4.07, SD = 1.67); [BCI > MoCap, *Z* = −1.841, *p* = 0.032]. In addition, the mean value for Q5 was significant between the two conditions showing a higher sensation of holding balls inside one’s own hand during the BCI session (M = 3.97, SD = 1.91) than the MoCap session (M = 3.03, SD = 1.83); [BCI > MoCap, *Z* = −2.063, *p* < 0.019]. There was not a significant difference between the two conditions for the other questions (Q4~Q9), although the mean values for Q4 [BCI (M = 2.39, SD = 1.50) > MoCap (M = 2.18, SD = 1.45); *Z* = −0.546, *p* = 0.292], Q6 [BCI (M = 3.70, SD = 1.76) > MoCap (M = 3.40, SD = 1.60); *Z* = −1.109, *p* = 0.133], and Q7 [BCI (M = 2.18, SD = 1. 70) > MoCap (M = 2.09, SD = 1.38); *Z* = −0.546, *p* = 0.292] were slightly higher in the BCI session than the MoCap session. The mean value of Q4 scores in both sessions was very low indicating that the sensation of having more than two hands was felt neither in the BCI session nor in the MoCap session. Similar results were acquired for Q7 scores, which asked about the sensation of one’s own hand plasticity, indicating that the subjects’ perception of the seen robot’s hand was rather a humanlike skin-covered hands than a plastic or rubber hand. On the other hand, although not significant, Q8 showed a higher mean value in the MoCap session (M = 3.06, SD = 1.64) than the BCI session (M = 3.70, SD = 1.55); [MoCap > BCI, *Z* = −1.585, *p* = 0.056], confirming that the attribution of the viewed hands to a person other than self was higher in the MoCap session than the BCI session. Lastly the subjects’ responses to Q9, which asked about the subject’s self-evaluated performance, showed high scores for both BCI (M = 5.61, SD = 1.32) and MoCap sessions (M = 5.64, SD = 1.54); [MoCap > BCI, *Z* = 0.005, *p* = 0.502] indicating that the positive bias of the classifier output could indeed result into a desirable self-evaluation of performance by the subjects in both sessions and therefore canceling out the effect of classifier performance on the intensity of illusion.

In respect to the SCR recordings (Fig. 2d), we only evaluated the response values of 28 participants, since five participants showed unchanged responses during the course of the experiment and therefore were excluded from the analysis. A Wilcoxon Signed-Rank test showed a significantly higher mean value for the BCI condition (M = 1.17, SD = 1.45) compared to the other MoCap condition (M = 0.67, SD = 1.12); [BCI > MoCap, Z = −2.781, *p* = 0.005].

In order to confirm the removal of movement and cancelation of proprioceptive signals in the BCI session, EMG signals were recorded from both left and right hands of each subject (Fig. 2b) prior to the experiment (Rest phase) and during the operational sessions. The experimenter monitored EMG signals particularly during the BCI session to ensure that no unconscious muscle engagement was involved during the motor imagery task, or in case of detection, could warn the subjects. Both online and offline monitoring of the recorded EMG activities in the BCI session confirmed that none of our subjects showed significant EMG changes that could correspond to movements or muscle contractions (see Methods). For each subject, the EMG peak amplitude in each trial of a session was collected and averaged in the corresponding session. The obtained values from all subjects were then averaged and compared to the EMG values in a Rest phase, recorded prior to the test sessions (Fig. 2e). A Wilcoxon Signed-Rank test showed that significantly stronger muscle contractions existed in the MoCap session (M = 113.97, SD = 55.53) compared to the Rest phase (M = 6.98, SD = 3.26); [Rest > MoCap, Z = −5.01, *p* = 2.32E-10], while the comparison between BCI session (M = 7.88, SD = 4.14) and Rest phase did not confirm a significant difference at the 0.05 level [Z = −1.69, *p* = 0.09].

At the end of the experiment, subjects were orally interviewed to express their opinion about each session. They were particularly asked three questions (Q1~Q3, Fig. 2f) in which they voted for the session with the stronger sensation of injection, level of ownership, and task feasibility. In regard to Q1 (injection), twenty-one subjects selected the BCI session, five subjects selected the MoCap session, and seven subjects expressed they had the same feeling of injection in both sessions, with a significant preference on the question [*X*^2^ (2, N = 33) = 13.82, *p* = 0.0001]. For Q2 (body ownership), twenty-five subjects voted for the BCI session whereas eight subjects selected the MoCap session [*X*^2^ (2, N = 33) = 29.64, *p* = 3.67E-7]. Lastly, in Q3 (task feasibility), seven subjects said the BCI condition was easier to operate, while twenty-six subjects voted in favor of the MoCap session [*X*^2^ (2, N = 33) = 32.91, *p* = 7.14E-8].

## Discussion

Both Q1 and SCR responses revealed that the operators’ reactions to a painful stimulus (injection) were significantly stronger in the BCI condition, where they controlled the robot’s hands by motor imagery, than the MoCap condition, where they made a robot’s hand grasp by grasping their own hands. Also, direct questions regarding motion attribution (Q2) and hand ownership (Q3) demonstrated a significantly higher sense of agency and body ownership in the BCI session. From the above results we can conclude that employment of a BCI-teleoperational interface for the control of a humanlike robot’s body could induce a stronger illusion of body ownership transfer in the operators than the conventional motion-capture interface in which operators were required to perform bodily motions.

An important factor which probably resulted in a weaker illusion in the MoCap condition compared to the BCI condition is the delay between the robot’s motions and the subject’s hand movements, which caused a mismatch between the visual feedback from the robot’s body and the proprioceptive updates from the subject’s body during the cognitive comparison process (Fig. 1a). Although the same delay existed in the BCI session between the onset of the motor imagery task until the robot performed the motion, and in fact the delay in the BCI session was longer than that of the MoCap session (Table 1), it did not weaken the illusion in the same way because the subjects did not move their own hands, thus mis-matching signals such as proprioceptive feedback were not present. The inhibiting effect of delay between sensory feedbacks has been previously reported in experiments of self-body recognition^12^,^13^,^14^,^18^. Shimada *et al*. showed that in RHI, a visual feedback delay longer than 300 ms could interrupt the visuotactile correlation and weaken the illusion until it diminished at 600 ms^13^. Similarly, our past experiments of robot’s arm operation, which employed a visuomotor integration, showed attenuation of BOT illusion due to a visual feedback delay^23^, although these results were slightly different from the RHI; the illusion effect still existed at 1 s delay, although weak and not significant to the control session.

But why does the BOT illusion for teleoperated androids occur even with a delay longer than that of the RHI threshold? The reason probably lies in the difference between the agency-driven illusions (visuomotor correlation) and sensory-driven illusions (visuotactile correlation). While agency is strongly related to body ownership (a body part you can control is perceived as your own body)^7^,^34^,^35^,^36^, the two senses are qualitatively different experiences^35^ that represent independent cognitive processes^8^ and recruit distinct neural networks^7^,^25^. Unlike ownership, agency can be extended to object-directed actions as long as the intention to move is present, even under temporal and spatial deviations^37^,^38^. Examples of such dissociation between agency and ownership have confirmed that when subjects controlled the motions of an incongruently positioned rubber hand or finger (spatial discrepancy during visuomotor integration), they felt agency for the motions but did not feel ownership for the body part^8^,^39^. Implementation of a systematic temporal delay in the visual feedback of movement has also revealed that the perception of agency can emerge by the visuomotor temporal recalibration, while the feeling of body ownership is disrupted^23^. Consistently with these results, it is suggested that during the robot teleoperation under delayed visual feedback (temporal discrepancy during visuomotor integration), the sense of agency that is the feeling of being the initiator and controller of the robot’s action remains intact. In such case, although subjects are capable of detecting the temporal discrepancy if the delay between the visual and proprioceptive feedback exceeds 200 ms^12^, their brain can establish a new mapping between motor intentions and sensory feedback. Throughout the new mapping, subjects adapt to the temporal discrepancy in the system by adding the delay component to the predictive model (forward model)^40^ that calculates the sensory outcome of the motor commands^18^,^41^ (Fig. 1a). Hence, if adequate time for adapting to the system is provided to the subjects, the recalibrated body representation modulates a sense of agency over the controlled body part. Nevertheless, as the delay between the visual feedback and subjects’ movements increases, the detection of mismatches between predicted sensory feedback (visual feedback) and actual sensory feedback (proprioceptive feedback) impedes the modulation of body ownership and only the sense of agency remains. In regard to this mechanism, some of our subjects reported such comments as: “When I moved my hand to move the robot, it seemed as if I am pushing a button to operate the robot” or “In the session that I moved my hand and the robot moved, it seemed that another person’s hand was moving in conjunction with my hand movements. As if a person next to me imitated my actions or raised his hand whenever I told him.” This clearly confirms the fact that subjects experienced agency for the robot’s motions in the MoCap session but they attributed the seen hands to somebody else rather than self. However we did not receive any comments regarding dissociation of agency and ownership in the BCI session. This shows that employment of a BCI-teleoperation interface, which cancels the mismatching proprioceptive signals, generates an interaction between agency and body ownership that is more robust to temporal delays.

A key element priming on the attribution of agency even with a long delay in the BCI session is the presence of strong motor intentions. The sense of agency is based on a mechanism that combines both internal motor commands and sensory outcomes to establish a relation between actions and their effects. Although agency can be modulated by extrinsic cues, the perceptual experience depends largely on the voluntary motor command itself^42^. Using active and passive movements, Tsakiris *et al*. probed the effect of temporal delay on sensation of agency and ownership^25^ when subjects moved their finger and watched synchronous and delayed projection of their hand (temporal discrepancy during visuomotor integration). The results obtained in this study suggest that although subjects reported a significantly higher sensation of agency and ownership in the active synchronous condition, the sensations did not completely vanish in the active delayed condition. However, when the experimenter passively performed the finger movement, the report of agency and ownership significantly dropped to rejection of the sensation in the delayed condition. In another study Wegner *et al*. reported that when subjects remained static and watched movements of another person’s hands placed in the same position as their own hands, receiving prior instructions about the movement could induce a sense of control over the viewed hands (vicarious agency)^43^. This all supports the notion that prior consistent thoughts about action play a dominant role in determining authorship, particularly when other sources of information such as tactile or proprioceptive feedback are unavailable or unclear.

Here, by canceling proprioceptive signals we refer to those afferent signals that carry information about muscle movements. The sense of proprioception generally refers to two kinds of sensation: that of static limb position and that of kinesthesia. In this experiment, although we did not remove the sense of limb position, we eliminated the updates in the kinesthesia component of proprioception using a BCI-teleoperational system (EMG was measured to ensure that unconscious muscle contractions were not present). Regardless of the subjects’ awareness over their own hand posture, our results suggest that the inducement of BOT illusion benefited from the removal of bodily motions (kinesthetic sense of proprioception) as long as the motor intentions and visual feedback remained. There are two elements that might have contributed to the redirection of subjects’ awareness from their own hands to the robot’s hands in the absence of movement; 1) the first-person perspective the subjects had over the robot’s hands. A recent study by Maselli and Slater showed that the sole effect of seeing a realistic virtual body in the same location and posture as one’s physical body can result into the illusion of owning the body with no need for further multisensory or sensorimotor inputs^44^. This makes the first-person perspective not only a critical factor but also a sufficient one for the onset of the body ownership illusion, where the contribution of additional inputs can produce a reinforcing effect when congruent and a suppressing effect when incongruent. In our experiment, the very humanlike shape and skin texture of the robot’s hands as well as the spatial congruency between the visual feedback and the existing position-related proprioceptive information could establish the first building blocks of the illusion. 2) The other factor that redirected subjects’ awareness from their own hands in the BCI session (and not in the MoCap session) is probably the sustained visual attention that subjects gave to the motor imagery task. Motor imagery is a difficult task that requires high concentration and increased attention. The selective attention that subjects paid to the task and resulted visual feedback could dominate the self-body awareness and the delay between the task onset (ball lighting) and robot’s motion^24^. On the other hand, there is evidence that signals related to efferent commands can contribute to the establishment of proprioception and sensation of position^30^,^31^. There is a possibility that during motor imagery, a small number efferent signals and accompanying proprioceptive feedback may be present at a level which cannot be measured with simple surface EMG. Nonetheless, the distinctive sense of effort involved in the BCI session seems to offer an important component for establishing the feeling of initiating the action and inducing the illusion. Indeed, the results of the follow-up interview (Fig. 2f) demonstrated that the majority of subjects chose the BCI session as evoking a stronger feeling of body ownership. When asked why, in addition to the disrupting delay in the MoCap session and the sensation of movement in their own hands, they referred to the task difficulty in the BCI session and the fact they were mostly focused on the robot’s motions. This is consistent with past studies that confirmed the domination of vision over other sensory modalities in coding the kinematic parameter of hand movements when a third sensory modality of proprioception is available^45^,^46^.

An interesting result in this experiment was the significant difference discovered in the scores of Q5 regarding the sensation of having balls in one’s hands. The higher mean value for the BCI session indicated the attenuation of cutaneous perception when proprioceptive signals were not updated. It was previously suggested that cutaneous receptors contribute to the proprioception by providing perceptual information about joint position and movement^47^. However, our results showed that in the absence of proprioceptive signals the cutaneous signals from the skin could be overridden by vision, providing evidence that the interaction between cutaneous signals and proprioception might be mutual. Previous works have already shown that the induction of ownership illusions for artificial body parts could alter the somatosensory perception in the participant’s real body. Moseley *et al*. showed that inducing RHI caused a decrease in skin temperature of the real hand and this effect was only specific to the stimulated limb^48^. In a psychometric study of embodiment with a detailed RHI questionnaire^49^, Longo and colleagues also found a component reflecting changes in the participants’ feelings about their own hand. They assumed that this component indicates a sense of disownership toward the real hand and accordingly called it ‘loss of own hand’. In another RHI study, Folgatti *et al*. measured the subjects’ reaction time to the tactile stimulus and found a slowing down of the tactile perception after the induction of the illusion^50^. The above studies support our finding in this experiment that the experience of ownership illusion toward a non-body object can regulate awareness of the physical self and attenuate the tactile acuity of the real body. This could be either due to the shift of attention toward the viewed body and the modulation of somatosensory perception by vision^5^ or due to the psychological disruption of the sense of ownership, which slows tactile processing of information from the real hand^48^.

The results of this experiment may initially look inconsistent with some of the previous works that highlighted the role of proprioceptive feedback in the inducement of agency-driven body ownership illusions^7^,^8^,^32^, however it should be noticed that in those experiments the illusion was compared between a synchronous and an asynchronous (not a consistent delay) motion paradigm and it was only induced for a finger of one hand. Therefore the amount of sensory input was limited in those experiments and the manipulation of subject’s sensation of proprioceptive inflow was more feasible. In another contradictory study that focused on the proprioceptive dominance in shaping the body representation in the space^51^, authors made a comparison between the importance of vision or proprioception in the online coding of body position in the space during a motor planning task that did not require a target movement. Their findings claimed that mental rotations were informed by proprioceptive inputs rather than vision, suggesting that the attribution of a hand based on position is specified mainly by proprioceptive information. A close look at their experimental paradigm shows that subjects watched pictures of a fake hand with angularly rotated postures while the subject’s real hand was hidden. However, in a few cases, some of the instructed rotations were difficult or implausible for a real human hand to execute (such as 240°). Therefore it was visually impossible for the subject to assign an absurd image of hand positioning to their previously established body frame. What we can infer is that their results mostly indicated the acceptance of a hand position to the subject’s body schema, rather than incorporation of the limb into their sensation of body ownership. Under such a definition, the proprioception component that is responsible for determination of limb orientation and position might play a dominant role in the attribution of fake body parts that were positioned congruently to the imaginary state. In our system, although we do not reject the effect of proprioception in the inducement of illusion, we were able to show that it is neither a dominant component nor an indispensable signal in the feedback loop. In fact, we suggest that in case of a mismatch between the sensory inputs, subjects would benefit from a direct interface that employs only visual feedback and eliminates the non-dominant proprioceptive signals.

Consistent with our results, recent studies have shown that spinal cord injury (SCI) patients who have lost proprioceptive sensations partially or completely are more prone to the experience of body ownership illusions^52^. In a series of RHI experiments with SCI patients, those who had injury at a lower level of the spinal cord and lost sensation and voluntary movement only in the lower body (intact sensation in the upper body and hands) revealed no alteration in the illusion compared to healthy subjects^53^, whereas those patients who had injury at the upper level of the cervical spinal cord and suffered from severe somatosensory and movement impairments in the hands, experienced a strong illusion of ownership for the rubber hand^54^. The difference between these two groups confirms our finding that in the absence of such core senses as proprioception, bodily representations can become uncertain and more amenable to changes, thus leading to the enhancement of the ownership illusion. This plasticity of brain also represents a significant potential for medical BCIs to employ the absence of sensations in physically impaired individuals as an opportunity to extend the sense of self and embodiment toward the artificial limbs and assistive prostheses.

Lastly, it could be speculated that the longer duration of the BCI training may have extended an enhancing effect on the adaptation of subjects to the temporal delays and therefore caused a stronger illusion in the BCI session compared to the MoCap session. In this experiment, subjects received a longer BCI training in order to first become familiar with the motor imagery task and second build up a level of confidence through the gradual bias of performance (See Methods). However, we do not think that the results of BOT scores could be affected by the frequency of BCI use because of two reasons: 1) the duration that the participants moved the robot during the training was equally 1 min for each (see Methods), and 2) subjects received an abstract visual feedback (not the realistic images of robot’s hands) during the additional BCI practice and hence did not experience BOT before the test sessions.

In conclusion, this study represents the practicality of BCI systems in generating an effective and powerful illusion of body ownership for humanlike robotic hands that are operated only by thoughts. Our results not only have promising impacts on the future development of medical BCIs such as neuroprosthetics that move and feel like one’s own limbs, but also provide new insights on how BCI-control of non-body objects may reshape the self-body representation for general users. We know from several neural and behavioral indications that the perception of self-body is not constant and even active tool-use can alter one’s body schema and extend the peripersonal space^55^,^56^,^57^. Our work suggests that the employment of BCIs for future device control can further make fundamental changes in the way we engage and interact with our surrounding world which, in long-term, can cause plastic changes in organization or properties of the neural circuitry. Lastly, it should be noted that the obtained results in this experiment are only valid when the BCI performance is positively biased to a favorable rate, and system errors are intentionally avoided. In reality, attaining such level of BCI performance with modern classifiers requires long training hours that are time-consuming and costly. Thus, the development of more powerful classifiers in the future is crucial to the application of BCI operational systems.

## Methods

This experiment was conducted with the approval of the Ethics Review Board of the Advanced Telecommunications Research Institute International (ATR), Kyoto, Japan. Approval ID: 14-601-3. All the methods carried out in the experiment were in accordance with the approved guidelines.

### Subjects

Thirty-three healthy participants (21 Male, 12 Female) in the age rang of 19~26 (M = 21.51, SD = 1.73), mainly university students, were selected for the experiment. All participants were naïve to the research topic and had normal or corrected to normal vision during the experiment. They signed a consent form in accordance with the regulations of ATR ethical committee in the beginning of the experiment and received a payment for their participation at the end.

### Motion-capture system

Subjects wore markers on their right and left middle fingers while performing grasp motions. A 3D motion-capture system consisting of three Motion Analysis Hawk Digital Cameras and EVaRT motion-capture software was used to track the marker position. In the beginning, subjects were instructed to lay their arms motionless on the chair-arm and keep their palms facing each other similar to the robot’s posture. The markers’ positions were set at this stage as the initial location. Subjects were then taught to perform a full grasp motion similar to that of the robot by only using four fingers (thumbs and arms remained still). They performed the grasp when the ball in robot’s hand was lighted, and held the grip until the light went off. Every time one of the markers was moved beyond a set threshold, a grasp command was sent to the robot’s corresponding hand.

### BCI system

Subjects wore an electrode cap and brain activities were recorded by 27 EEG electrodes installed over their primary sensori-motor cortex and g.USBamp biosignal amplifiers developed at Guger Technologies (Graz, Austria). The electrode placement was based on the 10–20 system (FT7, FC5, FC3, FC1, FCz, FC2, FC4, FC6, FT8, T7, C5, C3, C1, Cz, C2, C4, C6, T8, TP7, CP5, CP3, CP1, CPz, CP2, CP4, CP6, TP8). The reference electrode was placed on the right ear and the ground electrode on the forehead. The acquired data were processed online under Simulink/MATLAB (Mathworks) for real-time parameter extraction. This process included bandpass filtering between 0.5 and 30 Hz, sampling at 128 Hz, cutting off artifacts by a notch filter at 60 Hz, and adopting Common spatial pattern (CSP) algorithm to discriminate Event Related Desynchronization (ERD) and Event Related Synchronization (ERS) patterns associated with the motor imagery task^58^,^59^. Results were classified with weight vectors that weighed each electrode based on its importance for the discrimination task and suppressed the noise in individual channels by using the correlations between neighboring electrodes. During each right or left imagery movement, the decomposition of the associated EEG led to a new time series, which was optimal for the discrimination of two populations. The patterns were designed such that the signal from the EEG filtering with CSP had maximum variance for the left trials and minimum variance for the right trials and vice versa. In this way, the difference between the left and right populations was maximized and the only information contained in these patterns was where the EEG variance fluctuated the most during the comparisons between the two conditions. Finally, when the discrimination between left and right imaginations was made, the classification block outputted a linear array signal in the range of [−1, 1], where −1 denotes the extreme left and 1 denotes the extreme right.

### Experiment setup and procedure

Subjects wore a head-mounted display (Sony HMZ- T1)) through which they had a first-person view of the robot’s hands. Two randomly lighting balls were placed in front of the robot’s hands to give a cue for the motor imagery task during the operational sessions. Each participant performed the following two randomly conditioned sessions: 1) MoCap session, where subjects performed a grasp motion using their own right and left hand to control the robot’s corresponding hand, and 2) BCI session, where subjects performed a right or left motor imagery task and controlled the robot’s hands without actual motions. In both sessions, the trials lasted 7.5 s each. At second 2, an acoustic warning was given in the form of a “beep” to indicate the onset of the task and at second 3 the cue (lighting ball) was presented to the subject. From second 3.5 to 7.5, classifier results were sent to the robot in the form of motion commands. Throughout the experiment, identical blankets were laid on both the robot’s and subject’s legs so that the background view of the robot’s and subject’s bodies was the same.

### Training

Participants practiced robot control using each interface before starting the test sessions. For the MoCap session, subjects practiced robot control with their own grasp for 1 minute while watching the robot’s movement on a computer screen in front of them. In regard to the BCI session, subjects first performed a 40-trial non-feedback calibration session for the classifier setup and then a 60-trial feedback training session for motor imagery practice. In both calibration and motor imagery training sessions, participants watched the cue and the corresponding feedback on a computer screen in forms of a right/left arrow (for the cue) and a horizontal extending bar (for the feedback). The motor imagery training session was designed with an improving positive bias in the performance to convey the impression to the subjects that they had gradually improved and become able to control their brain activities. Such design was used so that the participants would not doubt the fake bias of the robot’s grasp in the following operational session. After completing the motor imagery training session, subjects practiced BCI-control of the robot’s hands for 1 minute while watching the robot’s movement on a computer screen in front of them.

### SCR recordings

Recorded signals were amplified by a multi-channel bio-signal amplifier (Polymate II AP216, TEAC Corp. Japan) at a sampling frequency of 1000 Hz. The peak amplitude of the responses to the injection was selected as a reaction value. Generally SCRs start to rise 1~ 2 seconds after a stimulus and end 5 seconds after that^3^. The moment at which the syringe appeared in the participant’s view was selected as the starting point for the evaluations, because some participants reacted to the syringe itself even before it was inserted into the robot’s hands as a result of the body ownership illusion^23^,^24^. Therefore, SCR peak values were sought within an interval of 6 seconds: 1 second after the syringe appeared in the participant’s view (1 second before it was inserted) to 5 seconds after the injection was actually made. In order to avoid the reaction to the surprise of the painful threat^3^ and make sure that SCR values exhibit a response due to the BOT illusion, each subject watched the injection to the robot’s hand inside HMD before the start of the test sessions and they were told that they might see the same scene during the operation. Participants who constantly showed baseline SCR values and did not exhibit changes during the course of the experiment were classified as non-responders and were excluded from the analysis. The inclusion criteria was any detectable peak larger than zero that could be observed due to the initial injection (the one before the start of the test sessions) or during the whole measurement. For those participants who had active SCR but showed no reaction to the stimulus, the peak response value within the reaction range was chosen for the analysis regardless of its sign (positive or negative).

### EMG recordings

Electromyographic signals were monitored during test sessions to especially confirm the elimination of proprioceptive feedback caused by muscle contractions in BCI session. Two pairs of EMG active electrodes were placed on the left and right forearm muscles (flexor carpi radialis) with the ground and reference electrodes attached to the wrist bones, the radius and the ulna. Recorded signals were amplified by a multi-channel bio-signal amplifier (Polymate II AP216, TEAC Corp. Japan). Before the appliance of the electrodes, subjects were asked to strongly make a grasp with each of their hands so that the experimenter could select the electrode position based on the muscle contractions. A conductive paste was applied to the electrode heads for a secure connection. For each subject, a 10-sec resting phase was recoded prior to the beginning of sessions. The peak value of EMG potentials in this phase was assigned as resting amplitude. In order to confirm the removal of movements in the BCI session, the mean EMG peak amplitudes in all trials of a session were calculated and the obtained mean for all subjects in each operational session was then compared to the resting amplitude.

## Additional Information

**How to cite this article**: Alimardani, M. *et al*. Removal of proprioception by BCI raises a stronger body ownership illusion in control of a humanlike robot. *Sci. Rep.* **6**, 33514; doi: 10.1038/srep33514 (2016).

## Figures and Tables

**Table 1:** Delay between the onset of task and robot motion in each operational session.

	Delay (ms)		
	Motion detection/Classification ~ Robot controller		Total
MoCap	≈0(marker tracking)	+ 200 ~ 600	200 ~ 600
BCI	≈500(motor imagery task)	+ 200 ~ 600	700 ~ 1100

Delay in the MoCap session was only due to the delay in the robot controller, which lasted 200 ~ 600 ms. Delay in the BCI session was longer because of an additional 500 ms between the onset of motor imagery task (cue presentation) and classifier output.
